# Bertolotti Syndrome: Surgical Treatment in a Middle-Aged Triathlete—A Case Report

**DOI:** 10.3390/healthcare13141712

**Published:** 2025-07-16

**Authors:** Julia Mahler, Alex Alfieri

**Affiliations:** 1 Neurosurgery, Cantonal Hospital of Winterthur, 8400 Winterthur, Switzerland; 2 Faculty of Biomedical Sciences, Università della Svizzera italiana, 6900 Lugano, Switzerland

**Keywords:** Bertolotti syndrome, lumbosacral transitional vertebra, processectomy, chronic low back pain, case report

## Abstract

**Background:** Bertolotti syndrome describes a painful lumbosacral transitional vertebra (LSTV) with a pseudoarticulation between an enlarged lateral process of the caudal lumbar vertebra (L5) and ilium or sacrum. It often presents with chronic lower back pain with or without radiculopathy. The current literature emphasizes Bertolotti as a differential diagnosis in young adults. However, it is presumably underdiagnosed in middle-aged and older patients. Treatment ranges from conservative treatment with physiotherapy, infiltration, and radiofrequency ablation to surgical interventions. **Case Description**: In this case illustration, we present the diagnostic and therapeutic challenges in a 48-year-old female triathlete with persistent left gluteal pain caused by Bertolotti syndrome. When conservative treatment with physiotherapy, infiltrations, thermocoagulation, and radiofrequency ablation of the pseudoarticulation failed, microsurgical reduction of the hypertrophic transverse process was performed. This minimally invasive intervention achieved satisfactory pain relief of at least 70% one year after surgery, allowing the patient to resume her athletic activities. **Conclusions**: Bertolotti syndrome should be considered a potential differential diagnosis in patients of all ages. Since many patients endure years of misdiagnosis, adequate treatment is crucial upon diagnosis. If conservative measures fail, surgical treatment such as “processectomy” or spinal fusion should be evaluated. This case follows the CARE reporting guidelines.

## 1. Introduction

Bertolotti syndrome was first described in 1917 by Bertolotti [1] and is a frequently overlooked pathology characterized by a symptomatic lumbosacral transitional vertebra (LSTV). This congenital anatomic anomaly consists of an enlarged lateral process of the caudal lumbar vertebra (L5), which forms a pseudoarticulation or fusion with the ilium or sacrum [2]. While the radiological presence of LSTV is relatively common and found in 4–30% of the general population [3], only few of these cases manifest clinically as Bertolotti syndrome with significant pain and disability. LSTV is classified according to Castellvi, distinguishing type I to IV, depending on the extent of the pseudoarticulation and fusion. Castellvi type I is the most common (42%) anomaly [2].

Leading symptoms are chronic lower back pain (LBP) and/or (pseudo-)radicular symptoms resulting from mechanical stress, degeneration of the pseudoarticulation, and/or irritation of the exiting L4 nerve root [2,4]. Bertolotti syndrome remains a diagnostic challenge, frequently overlooked in favor of more common causes of back pain. In 2006, Quinlan et al. [5] indicated a higher prevalence in younger patients, making it less likely to be considered a differential diagnosis in middle-aged and older patients. A delay of up to seven years in diagnosis and treatment leads to prolonged patient suffering [6].

First-line treatment of Bertolotti syndrome includes conservative treatment with physiotherapy, infiltration, and radiofrequency ablation [2,7]. When conservative treatment fails, surgical resection of the enlarged transverse process (“processectomy”) or spinal fusion are performed [4,6,8]. There are no large-scale studies investigating the failure rate of conservative treatment nor any guidelines recommending the switch from conservative to surgical treatment based on concrete parameters.

We present a 48-year-old, highly active patient and emphasize that Bertolotti syndrome can occur in middle-aged, very athletic and active patients. For such patients in particular, the reduction in quality of life due to pain, often inadequately treated, is very significant. Future studies should include patients from all age groups. The discussion includes diagnostic challenges, limitations of conservative treatment, and outcomes of surgical treatment. This case follows the CARE reporting guidelines.

## 2. Case Presentation

**Figure 1:**
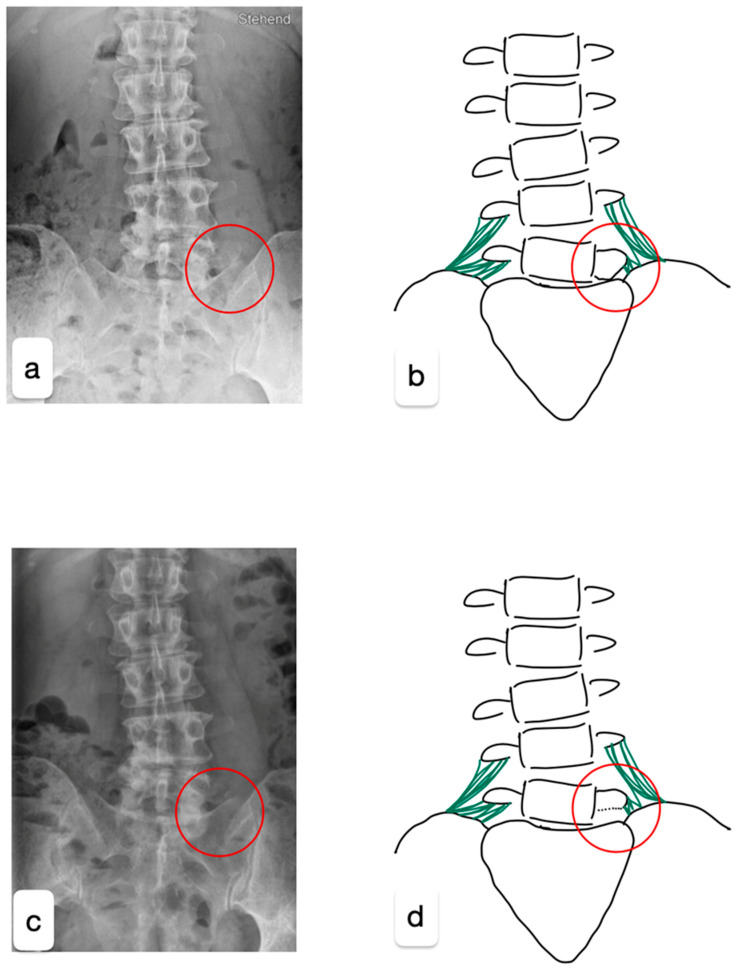
X-rays in the anteroposterior projection before (**a**) and after the surgery (**c**), supplemented by a graphical simplification of the imaging (**b**,**d**). The red circle highlights the location of the LSTV. The surgery aimed to release the existing connections between the pelvis and the malformed transverse process of L5 while preserving the iliolumbar ligament (green) through microsurgical techniques to maintain stability.

A 48-year-old healthy female triathlete presented to our outpatient clinic with pain in the left gluteal region persisting for two years, especially during sporting activities. No relevant past medical history. Neurological examination of the patient was unremarkable, showing normal flexibility and mild tenderness over the left sacroiliac joint. Magnetic resonance imaging and computed tomography revealed multisegmental degenerative changes with a right convex scoliosis, multisegmental disc extrusion without relevant stenosis, osteochondrosis L2/3, L4/5, and L5/S1, facet joint arthrosis L3/4, and left-sided LSTV (Castellvi type 1a) (Figure 1a,b).

Prior to our consultation, the conservative treatment consisted of diagnostic–therapeutic infiltration of the L4-S1 levels and the sacroiliac joint, providing only temporary relief. Pathologies of the spinal canal, facet joints, and sacroiliac joints therefore appeared less likely as a differential source of pain. Repeated infiltrations of the L5/S1 pseudoarticulation later confirmed the diagnosis of Bertolotti syndrome but failed to achieve pain relief lasting for more than three weeks. Additional conservative treatments, including physiotherapy, manual therapy, chiropractic interventions, thermocoagulation, and radiofrequency ablation of the pseudoarticulation, were also ineffective in the long term. The patient continued to experience significant pain during physical activities such as running and swimming, resulting in a major impact on her quality of life.

**Figure 2:**
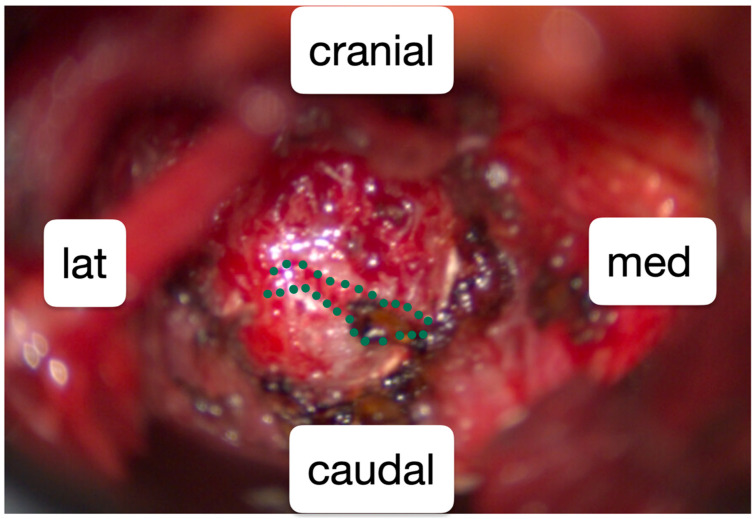
Intraoperative view of the reamed pseudoarticulation (green), which had formed between the pelvis and the transverse process L5 on the left.

As the conservative treatment was exhausted, surgical reduction of the left L5 transverse process was performed. For the surgery with general anesthesia, the patient was positioned in a prone position on a Wilson frame. The L5/S1 neoarticulation was visualized using X-ray control (anterior–posterior and lateral beam path). Following a paramedian skin incision on the left L5/S1 level and dissection of the fascia and paravertebral musculature, a Caspar retractor was inserted. The caudal part of the transverse process L5 was reduced microsurgically using a 6 mm diamond drill and bone punch (Figure 1 and Figure 2). Strong adhesions to the pelvis were exposed and the L4 nerve root could be visualized.

Following a three-night stay, the patient was discharged home without any peri- or postoperative complications. Postoperative follow-up visits up to six months showed a satisfactory resolution of her chronic gluteal pain, allowing her to resume marathon training. At the most recent consultation, a year after surgery, the patient reported a pain reduction of at least 70% compared to before the surgery. This case highlights the effectiveness of surgical intervention for refractory Bertolotti syndrome, especially in active patients with significant functional impairment.

## 3. Discussion

Bertolotti syndrome describes a symptomatic LSTV in which the congenital enlarged lateral process of the caudal lumbar vertebra (L5) forms a pseudoarticulation with the ilium or sacrum [2]. First described by Bertolotti in 1917 [1], this condition is often overlooked or misinterpreted as a mere radiological anomaly rather than as a clinically significant source of pain. Uni- and bilateral radiological anomalies of the L5 transverse process are classified according to Castellvi [9]. LSTV Castellvi type I is the most common anomaly [2], as was found in the presented case. Bertolotti syndrome commonly presents as uni- or bilateral LBP, sometimes accompanied by (pseudo-)radicular pain. These symptoms result from L4 nerve compression (radicular symptoms) or degeneration of the pseudoarticulation (pseudoradicular symptoms) [2,4]. The L4 nerve root, located near the hypertrophic L5 transverse process, is particularly vulnerable to irritation [4]. Altered biomechanics from LSTV may predispose individuals to earlier degenerative spinal changes [10], possibly due to asymmetry between the lumbar vertebrae and sacrum [7]. Bertolotti syndrome is the clinical correlate of the congenital, usually asymptomatic, LSTV.

The age at diagnosis of Bertolotti syndrome varies significantly across different published studies. Quinlan et al. [5] reported a mean age of 32.7 years for patients with radiologically and clinically confirmed Bertolotti syndrome. In contrast, the presented patient is unique due to the relatively late onset of symptoms associated with congenital LSTV (Bertolotti syndrome). Perhaps her sporting activities delayed pain until later in life. Studies on protective factors such as physical constitution and sporting activities are needed to evaluate which and why patients with congenital LSTV develop Bertolotti syndrome. In contrast, Il Ju et al. [4] reported a mean age of 55.9 years for patients with Bertolotti syndrome requiring surgery, and Ravikanth et al. [11] found no statistically significant difference in age distribution between patients with radiologically confirmed Bertolotti syndrome and those with unspecific LBP, highlighting the variability in presentation.

Patients with Bertolotti syndrome often endure LBP with or without radicular symptoms for several years before receiving a diagnosis; Santarvirta et al. [6] reported an average of seven years from onset of LBP to surgical intervention. This time is marked, besides severe pain and reduced quality of life, by many (potentially invasive) examinations, painful procedures, and, depending on the country, healthcare expenses and lost income. Treatment of Bertolotti syndrome ranges from conservative first-line treatment with physiotherapy, injections, and radiofrequency ablation to surgical intervention including resection of the enlarged transverse process (“processectomy”) or spinal fusion in refractory cases [2,7].

Holm et al. [8] reviewed 79 cases treated with either infiltrations or surgery, of which 33 patients underwent transverse process resection, 8 had spinal fusion, and 7 had laminectomy. Santarvirta et al. [6] compared surgical and conservative treatments, finding that surgery resulted in slightly lower pain scores (1.9 vs. 2.5). However, they noted that only four out of six patients with a positive preoperative infiltration response achieved significant postoperative improvement. This emphasizes the importance of a reliable preoperative diagnosis. In our case, particularly with a highly active patient, we opted for the less invasive surgical therapy and thus for a sole reduction of the transverse process instead of spinal fusion with potential long-term complications such as adjacent segment disease.

In the end, Bertolotti syndrome is a complex clinical picture that requires a personalized interdisciplinary conservative and surgical treatment. If conservative treatment fails, even in active patients, surgical treatment should be evaluated in an interdisciplinary setting and recommended if deemed appropriate.

This case report has limited generalizability as it describes only a single highly active middle-aged patient. Further studies are needed to investigate the prevalence and adequate treatment in (active) middle-aged and older patients.

## 4. Conclusions

We presented a single case of a highly active middle-aged patient suffering from therapy-refractory Bertolotti syndrome with extensive conservative therapy.

Bertolotti syndrome is a significant yet often overlooked cause of chronic lower back and gluteal pain in patients with LSTV. This case highlights the importance of considering Bertolotti syndrome in the differential diagnosis of chronic LBP in patients of all ages. Though traditionally associated with younger demographics [5], symptomatic cases have been reported in patients as old as 65 years [12]. The prolonged diagnostic delays seen in many patients, including this case, underscore the importance of imaging studies and targeted infiltrations for accurate diagnosis. First-line conservative treatment may fail in highly active patients, making surgical intervention necessary. As this is a single case report, larger-scale studies are needed to investigate the value of surgical treatment in active and/or elderly patients.

For this patient, microsurgical reduction of the caudal part of the hypertrophic transverse process after exhaustion of conservative treatment proved to be the definitive treatment. This case illustrates the need for tailored treatment and considering surgery in case of therapy-refractory Bertolotti syndrome. Further research focusing on long-term outcomes and the development of standardized diagnostic and treatment guidelines would improve patient care for this often unrecognized condition.

## Data Availability

The original contributions presented in this study are included in the article. Further inquiries can be directed to the corresponding author.

## Abbreviations

The following abbreviations are used in this manuscript:

LBP	lower back pain
LSTV	lumbosacral transitional vertebra
